# Trajectories of maternal depressive and anxiety symptoms from pregnancy to five years postpartum and their prenatal predictors

**DOI:** 10.1186/s12884-019-2177-y

**Published:** 2019-01-14

**Authors:** Asma Ahmed, Angela Bowen, Cindy Xin Feng, Nazeem Muhajarine

**Affiliations:** 1 Department of Epidemiology, Biostatistics and Occupational Health, Faculty of Medicine, McGill University, Purvis Hall, 1020 Pine Avenue W, Room 27, Montreal, Quebec H3A 1A2 Canada; 2 College of Nursing, University of Saskatchewan, 104 Clinic Place, Health Sciences Building, Room 4246, Saskatoon, Saskatchewan S7N 2Z4 Canada; 3 School of Public Health, University of Saskatchewan, 104 Clinic Place, Health Sciences Building, Room 3338, Saskatoon, Saskatchewan S7N 2Z4 Canada; 4 College of Medicine, University of Saskatchewan, 104 Clinic Place, Health Sciences Building, Room 3246, Saskatoon, Saskatchewan S7N 2Z4 Canada

**Keywords:** Longitudinal trajectories, Maternal depression, Maternal anxiety, Mood disorders, Risk factors

## Abstract

**Background:**

Maternal depression and anxiety have distinct constellations of symptom trajectories, which are associated with factors that may vary between different groups of women. The aim of this study was to identify subgroups of women who exhibit unique longitudinal trajectory patterns of depressive and anxiety symptoms from pregnancy to 5 years postpartum and the antenatal predictors associated with these maternal groups.

**Methods:**

The study used a longitudinal data collected from 615 women in Saskatchewan from pregnancy to 5 years postpartum. Semiparametric group-based models were used to identify latent maternal depressive and anxiety trajectory groups. Multinomial logit models were then used to assess the association between maternal characteristics and the identified latent trajectory groups.

**Results:**

We identified four trajectory groups of maternal depressive symptoms: low-stable (35%); moderate-stable (54%); moderate-increasing (5%); and high-decreasing (6%), and three trajectory groups of maternal anxiety symptoms: very low-stable (13%); low-stable (58%); and moderate-stable (29%). We also identified several risk factors, most notably history of depression and stress, that were significantly associated with these trajectories.

**Conclusion:**

History of depression and increased stress are significant risk factors that can be identified during regular perinatal visits; therefore, clinicians should inquire about these risk factors to identify women at high risk of ongoing depression or anxiety.

**Electronic supplementary material:**

The online version of this article (10.1186/s12884-019-2177-y) contains supplementary material, which is available to authorized users.

## Background

Depression and anxiety disorders are highly prevalent among childbearing women, with rates as high as 30% [1, 2]. Maternal depression and anxiety are associated with poor health outcomes for the woman and her entire family [3, 4], which may have serious implications for the child’s developmental and psychological outcomes if untreated [5, 6]. Moreover, chronic depression affects long-term maternal health, with increased psychiatric morbidity (most notably more frequent and severe depressions) as well as physical and cognitive decline [7].

There is a growing evidence that maternal depressive and anxiety symptoms are heterogeneous, highly diversified with their onset, course, duration, and severity [8–12]. Nandi et al., reviewed population-based studies of depression and anxiety trajectories and concluded that research in this area is still in its infancy; nonetheless, they found studies which confirm distinct groups of symptom trajectories for depression and anxiety (i.e., clusters of women who follow similar symptom patterns s over time), and that these trajectories are associated with risk factors that may vary between groups [9]. A more recent systematic review of perinatal depressive symptom trajectories found a similar pattern of depressive trajectories across studies and emphasized the need for further research within different settings [13].

Different methods have been used to model the developmental trajectories of maternal mental health symptoms over time. Both longitudinal mixed-effects and latent growth curve models model individual variabilities of these symptoms over time using a single growth curve, assuming that all individuals belong to the same underlying population [13]. Hence, these models may oversimplify the underlying complexity and heterogeneity of symptoms [13]. Alternatively, group-based trajectory modeling can be used to identify clusters of individuals who follow a similar evolution of behaviour [14]. This method is particularly important in the case of maternal depression and anxiety, as it allows for capturing the diversity of these symptoms in terms of onset, course, timing, and severity [15]. Furthermore, these models do not require prior information on the number and shape of groups, and they allow the magnitude and direction of change of depressive or anxiety symptoms to vary between different trajectories [14].

Previous research on the trajectories of maternal depression has examined the period between late pregnancy up to 1 to 2 years postpartum [16–18]. Very few studies have examined maternal anxiety trajectories with most up to 1–2 years postpartum, with mixed results [16, 17, 19]. These studies provide evidence for longitudinal trajectories of maternal depression and anxiety; but, findings vary with the study population, location, and start and length of follow-up. Moreover, whereas the predictors for maternal depression and anxiety are well documented, there remains a paucity of research that links longitudinal trajectories of maternal depression and anxiety to their risk factors. This is particularly important as some risk factors may be associated with certain subgroups of women with maternal depression or anxiety, which would allow for targeted interventions. Early intervention directed towards at high risk women has been shown to reduce the risk of developing major depression [20]. Thus, it is essential to explore the trajectories of maternal depressive and anxiety symptoms beyond the perinatal period not only to recognize which women target by early mental health interventions but also to identify modifiable risk factors.

This study sought to identify maternal depressive and anxiety symptoms’ trajectory groups and their antenatal predictors and answer the following questions: 1. What are the distinct trajectory patterns for maternal depressive symptoms from pregnancy to 5 years postpartum, and what are the antenatal predictors associated with these trajectory groups? and 2. What are the distinct trajectory patterns for maternal anxiety symptoms from pregnancy to 5 years postpartum, and what are the antenatal predictors associated with these trajectory groups?

## Methods

### Sample

This study uses data from the Feelings in Pregnancy and Motherhood Study and follow up study of child and maternal outcomes (FIP), a longitudinal epidemiological study of maternal depression and associated factors [21, 22]. In brief, in 646 women recruited from the community. Women were eligible to participate in the study if they were: 1. within the first 20 weeks of pregnancy, 2. able to speak English, and 3. residing in one of two regional health authorities in Saskatchewan (Saskatoon Health Region and Five Hills Health Region). Data was collected via face-to-face individual interviews by trained research assistants five times; early pregnancy (17.4 +/− 4.9 weeks gestation) and late pregnancy (30.6 +/− 2.7 weeks gestation), once in early postpartum (4.2 +/− 2.1 weeks), and again at 36 and 60 months postpartum. A detailed description of the FIP study is published elsewhere [21, 22].

Missing data was managed using the PROC TRAJ maximum likelihood estimation to estimate model parameters when there are at least two observations per individual [8, 23]. Nagin indicates that it is reasonable to exclude cases with very incomplete assessment histories [14], which was also done by other researchers who used the same methodology [8]. For this study, we excluded cases with more than three missing values, and thus our sample included 615 participants at time 1 (early pregnancy), 601 women at time 2 (late pregnancy), 592 women at time 3 (early postpartum), 337 women at time 4 (36 months postpartum), and 308 women at time 5 (60 months postpartum). Written consent was informed and ethical approval was received from the Office of Research Ethics at the University of Saskatchewan.

### Measures

#### Antenatal predictors

Selection of antenatal predictors was based on those reported in the literature, such as age, marital status, ethnicity, education, employment, income, parity, whether the pregnancy was planned or not, level of satisfaction with their relationship with the partner, if they have one, any history of depression, as well as behavioural factors (smoking, alcohol, recreational drug use, and exercise level). Social support was measured by asking participants about people who provide them with emotional support. Responses were summed into a summary variable that indicates the level of social support as low-level (0–1 support) and high-level of support (two or more supports). Women were asked to indicate sources of stress from a list of stressors, their responses were combined into one summary variable that was dichotomized as low stress level (0–2 stressors) or high stress level (more than two stressors).

#### Outcome variables

##### Perinatal depressive symptoms

The Edinburgh Postnatal Depression Scale (EPDS) was used to assess women for depressive symptoms [24]. The EPDS is one of the most commonly validated screening tools for detection of perinatal depression with sensitivity of 59–100%, specificity of 49–100%, and good internal consistency (Cronbach’s Alpha> 0.80) [25, 26]. It is a 10-item self-rated questionnaire, and responses are reported on a Likert scale from zero to three with a maximum score of 30.

##### Perinatal anxiety symptoms

The three-item anxiety subscale (EDPS-A) was used to screen for anxiety symptoms [27]. Bowen and colleagues have confirmed an EPDS anxiety subscale (Items 3–5) during pregnancy (Cronbach’s alpha coefficient = 0.71) [28], while Ross et al., confirmed the same factors in postpartum women [29]. Responses are reported on a Likert scale from zero to three with a maximum score of nine [30].

### Data analysis

The semiparametric, group-based approach for modeling developmental trajectories [14] was used to identify trajectories of maternal depressive symptoms based on their total EPDS scores and anxiety symptoms based on their EPDS-A scores from early pregnancy to 5 years postpartum. The PROC TRAJ procedure in SAS was used to estimate group-based trajectories models [31, 32]. In our study, we chose to use the censored normal distribution (CNORM) distribution as depression and anxiety scores tend to cluster at their respective minimum values depicted as a right-skewed distribution.

A two-stage model selection strategy was used to find the optimum number of groups and shape of trajectories that best fit the data [14]. In the first stage, we started to test models that consisted of two-six groups with cubic degree polynomial, guided by previous literature [8, 10, 16, 33]. Once the number of groups was identified based on a series of model selection criteria, i.e., Bayesian Information Criterion (BIC), Bayes factor, and the probability of being the correct model, a backward elimination method was used to select the order of trajectories. Non-significant cubic, quadratic, and linear terms were removed consecutively until all terms in the model were significant. Theoretical considerations such as the expected number and shape of trajectories, as well as the interpretability of these trajectories, were also considered [8]. To check whether the model fits the data well, several model diagnostic methods were used, including the average posterior probability of assignment, the odds of correct classification, the estimated group probability versus proportion of sample assigned to the group, and the confidence intervals for group membership probability.

Due to significant attrition, especially at the 36th and 60th months postpartum, we included the dropout statement extension in the PROC TRAJ SAS procedure to account for missing data [23]. We then compared models with and without the dropout statement to check the magnitude of change in the trajectory shape parameter estimates, as well as group membership probabilities.

Multinomial regression models were then used to assess the effects of various maternal characteristics at baseline on the probability of belonging to a specific trajectory group compared to a reference group [14, 34]. All analyses were performed using SAS software version 9.4.

## Results

Over 50% of participants were primiparous, and most were Caucasian women living in a stable relationship with a moderately high socioeconomic status (see Table 1).

**Table 1:** Sociodemographic, psychosocial, and behavioral characteristics of FIP cohort study participants (*n* = 615) at different latent trajectory groups of maternal depression and anxiety symptoms [n (%)]

		Depression trajectories					Anxiety trajectories			
Maternal characteristics	Total (*n* = 615)	Low-stable (*n* = 215)	Moderate-stable (*n* = 332)	Moderate-increasing (*n* = 32)	High-decreasing (*n* = 36)	*p*-value	Very low-stable (*n* = 80)	Low-stable (*n* = 357)	Moderate-stable (*n* = 178)	*p*-value
Mother’s age						0.025				< 0.001
< 25 years	96 (15.6)	27 (12.6)	50 (15.1)	8 (25.0)	11 (30.6)		7 (8.8)	44 (12.3)	45 (25.3)	
≥ 25 years	519 (84.4)	188 (87.4)	282 (84.9)	24 (75.0)	25 (69.4)		73 (91.3)	313 (87.7)	133 (74.7)	
Parity						0.438				0.850
0	327 (53.3)	121 (56.3)	171 (51.6)	19 (59.4)	16 (44.4)		42 (52.5)	187 (52.5)	98 (55.1)	
≥ 1	287 (46.7)	94 (43.7)	160 (48.3)	13 (40.6)	20 (55.6)		38 (47.5)	169 (47.5)	80 (44.9)	
Education						0.022				0.147
< grade 12	24 (3.9)	5 (2.3)	12 (3.6)	2 (6.3)	5 (13.9)		1 (1.3)	12 (3.4)	11 (6.2)	
≥ grade 12	590 (96.1)	209 (97.6)	320 (96.4)	30 (93.8)	31 (86.1)		79 (98.8)	344 (96.6)	167 (93.8)	
Ethnicity						0.004				0.036
Caucasian	522 (85.0)	193 (89.8)	280 (84.6)	25 (78.1)	24 (66.7)		73 (91.3)	308 (86.3)	141 (79.7)	
Non-Caucasian	92 (15.0)	22 (10.2)	51 (15.4)	7 (21.9)	12 (33.3)		7 (8.8)	49 (13.7)	36 (20.3)	
Marital status						0.001				0.029
Non-partnered	57 (9.3)	10 (4.7)	33 (9.9)	4 (12.5)	10 (27.8)		4 (5.0)	28 (7.8)	25 (14.0)	
Partnered	558 (90.7)	205 (95.4)	299 (90.1)	28 (87.5)	26 (72.2)		76 (95.0)	329 (92.2)	153 (86.0)	
Employment						0.001				0.050
Yes	487 (79.5)	185 (86.1)	259 (78.3)	22 (68.8)	21 (60.0)		68 (85.0)	289 (81.2)	130 (73.5)	
No	126 (20.6)	30 (14.0)	72 (21.8)	10 (31.3)	14 (40.0)		12 (15.0)	67 (18.8)	47 (26.6)	
Income						< 0.001				< 0.001
< $20,000	75 (12.5)	14 (6.5)	40 (12.4)	5 (16.1)	16 (45.7)		2 (2.5)	36 (10.3)	37 (21.6)	
20-$40,000	109 (18.1)	33 (15.4)	64 (19.9)	8 (25.8)	4 (11.4)		15 (18.8)	57 (16.2)	37 (21.6)	
40-$60,000	129 (21.4)	41 (19.2)	76 (23.6)	6 (19.4)	6 (17.1)		15 (18.8)	70 (19.9)	44 (25.7)	
> $60,000	289 (48.0)	126 (58.9)	142 (44.1)	12 (38.7)	9 (25.7)		48 (60.0)	188 (53.6)	53 (31.0)	
Past depression						< 0.001				< 0.001
Yes	217 (35.3)	35 (16.3)	136 (41.0)	18 (56.3)	28 (77.8)		12 (15.0)	99 (27.7)	106 (59.6)	
No	398 (64.7)	180 (83.7)	196 (59.0)	14 (43.8)	8 (22.2)		68 (85.0)	258 (72.3)	72 (40.5)	
Stress level						< 0.001				< 0.001
Low (0–2)	362 (59.4)	170 (80.6)	174 (52.7)	14 (43.8)	4 (11.1)		66 (84.6)	223 (63.0)	73 (41.2)	
High (> 2)	247 (40.6)	41 (19.4)	156 (47.3)	18 (56.3)	32 (88.9)		12 (15.4)	131 (37.0)	104 (58.8)	
Support level						0.043				0.105
Low (0–1)	104 (16.9)	25 (11.6)	63 (19.0)	6 (18.8)	10 (27.8)		8 (10.0)	59 (16.6)	37 (20.8)	
High (> 1)	510 (83.1)	190 (88.4)	268 (81.0)	26 (81.3)	26 (72.2)		72 (90.0)	297 (83.4)	141 (79.2)	
Relationship satisfaction						< 0.001				0.009
Not satisfied	75 (12.3)	14 (6.5)	38 (11.6)	6 (18.8)	17 (48.6)		7 (8.8)	35 (9.9)	33 (18.8)	
Satisfied	536 (87.7)	201 (93.5)	291 (88.5)	26 (81.3)	18 (51.4)		73 (91.3)	320 (90.1)	143 (81.3)	
Planned pregnancy						0.001				0.030
Yes	366 (59.5)	147 (68.4)	190 (57.2)	16 (50.0)	13 (36.1)		55 (68.8)	218 (61.1)	93 (52.3)	
No	249 (40.5)	68 (31.6)	142 (42.8)	16 (50.0)	23 (63.9)		25 (31.3)	139 (38.9)	85 (47.8)	
Smoking						< 0.001				0.011
No or quit	548 (89.3)	203 (94.4)	293 (88.5)	27 (84.4)	25 (69.4)		76 (95.0)	324 (90.8)	148 (83.6)	
Yes	66 (10.8)	12 (5.6)	38 (11.5)	5 (15.6)	11 (30.6)		4 (5.0)	33 (9.2)	29 (16.4)	
Alcohol						0.321				0.402
No or quit	573 (93.2)	202 (94.0)	309 (93.1)	31 (96.9)	31 (86.1)		77 (96.3)	329 (92.2)	167 (93.8)	
Yes	42 (6.8)	13 (6.1)	23 (6.9)	1 (3.1)	5 (13.9)		3 (3.8)	28 (7.8)	11 (6.2)	
Drugs						0.310				0.250
No or quit	596 (97.1)	208 (97.2)	324 (97.6)	31 (96.9)	33 (91.7)		80 (100.0)	343 (96.4)	173 (97.2)	
Yes	18 (2.9)	6 (2.8)	8 (2.4)	1 (3.1)	3 (8.3)		0.00 (0.0)	13 (3.7)	5 (2.8)	
Exercise						0.103				0.248
Never or occasional	284 (46.2)	90 (41.9)	155 (46.7)	16 (50.0)	23 (63.9)		33 (41.3)	160 (44.8)	91 (51.1)	
Regular	331 (53.8)	125 (58.1)	177 (53.3)	16 (50.0)	13 (36.1)		47 (58.8)	197 (55.2)	87 (48.9)	

### Maternal depression trajectory groups

**Fig. 1:**
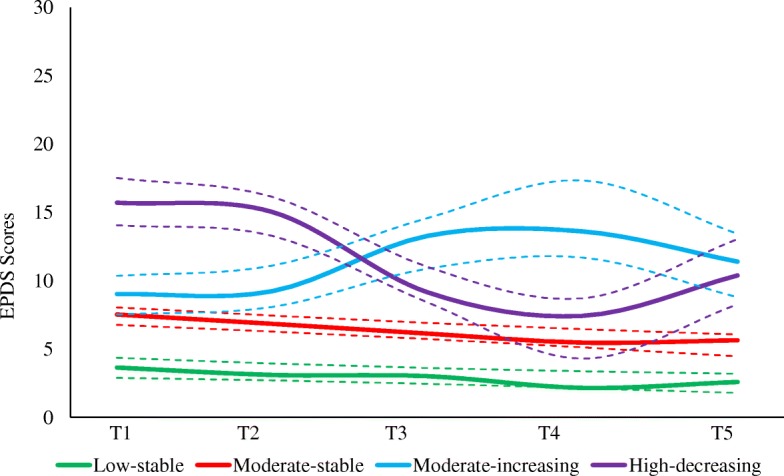
Trajectory groups of maternal depression symptoms of women from the FIP cohort study (*n* = 615). The solid lines represent the observed trajectory groups and the dashed lines represent 95% confidence intervals. T1-T5 are the 4th month of pregnancy, the 7th month of pregnancy, 1 month postpartum, 36 months postpartum, and 60 months postpartum respectively

Candidate models containing two to six groups were analyzed. The BIC score increased from the two-group to the four-group model, and then it started to decrease as further groups were added. The Bayes Factor showed strong evidence in support of the four-group model compared to the three-group and five-group model; the probability of being the correct model was the highest for the four-group model (0.984). Therefore, we chose the four-group model as the best fitting and most parsimonious model (see Additional file 1 for model fit indices). Figure 1 depicts the four maternal depression groups with 95% confidence limits. The average posterior probability ranged from 0.83 for the moderate-increasing group to 0.91 for the high-decreasing group (mean = 0.86), indicating a very good model fit. In addition, the model met the other three indicators of model adequacy (Additional file 2).

The first depression group “low-stable” (*n* = 215, 35.0%) included women whose EPDS scores were consistently low throughout the follow-up period. Women in the largest group “moderate-stable” (*n* = 332, 54.0%) reported a moderate level of depressive symptoms across the period of follow-up that minimally decreased over time. More fluctuations were seen in the other two groups (moderate-increasing and high-decreasing), as evident from the significant cubic term for both groups. Depression scores for women assigned to the moderate-increasing group (*n* = 32, 5.2%) were slightly higher than those of the moderate-stable group during pregnancy; however, their EPDS scores increased significantly postpartum. Women in the high-decreasing group (*n* = 36, 5.9%) experienced high levels of depressive symptoms during pregnancy which started to decrease gradually after giving birth, except for a slight increase between the third and fifth year of follow-up (EPDS scores are summarized by time and depressive trajectory group in Additional file 3).

Comparison of the basic model and the extended model that accounts for participants’ attrition revealed that parameter estimates and group membership probabilities for the low-stable and moderate-stable groups were almost identical between both models. However, the estimates for the linear, quadratic, and cubic terms, as well as group membership probabilities of the other two groups were different (see Additional file 4).

### Maternal anxiety trajectory groups

**Fig. 2:**
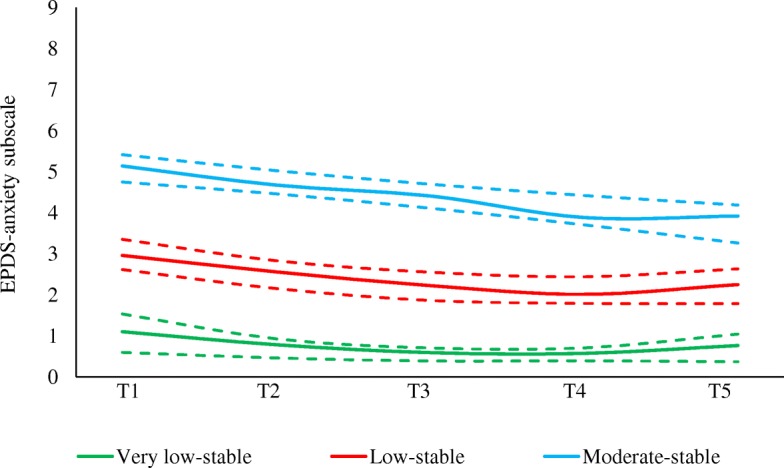
Trajectory groups of maternal anxiety symptoms of women from the FIP cohort study (*n* = 615). The solid lines represent the observed trajectory groups and the dashed lines represent 95% confidence intervals. T1-T5 are the 4th month of pregnancy, the 7th month of pregnancy, 1 month postpartum, 36 months postpartum, and 60 months postpartum respectively

To determine the best number of anxiety groups, we compared BIC values of the two-group to six-group models. The BIC score increased from the two-group model to the three-group model, but they started to decrease as further groups were added (see Additional file 5). Hence, we concluded the three-group model as the best fitting and most parsimonious model. Figure 2 illustrates the three maternal anxiety groups with their 95% confidence limits. The average posterior probability ranged from 0.87 to 0.88 for the very low-stable group (mean = 0.87), indicating a very good model fit. Other model fit diagnostics also indicated a good model fit (Additional file 6).

The first and the smallest group “very low-stable” (*n* = 80, 13.0%) was composed of women whose anxiety symptoms were the lowest of the sample and were relatively stable over the whole period of follow-up. More than half the sample (*n* = 357, 58.1%) belonged to the second group “low-stable”, and their anxiety scores were fairly constant at levels that were higher than those of the very low-stable group. The third group “moderate-stable” included women with moderate to high anxiety scores throughout the period of follow-up, but with a slight decrease over time; one-third of the sample (*n* = 178, 29.0%) fell into this group. EPDS-A scores are summarized by time and anxiety trajectory group in Additional file 7. Comparison of the model with and without dropout extension revealed that trajectory shape parameter estimates, as well as group membership probabilities for both models, were almost identical (refer to Additional file 4).

### Multinomial regression

For depression trajectory groups, risk factors of membership in the moderate-stable compared with the low-stable group included a history of depression, high stress level, and non-Caucasian ethnicity. Compared to the low-stable group, membership in the moderate-increasing group was associated with a history of depression and high stress level. For the high-decreasing group, significant associations compared to the low-stable group included a history of depression, high stress level, being a tobacco user, and non-Caucasian ethnicity (see Table 2).

**Table 2:** Sociodemographic, psychosocial, and behavioral predictors of the latent trajectory group membership for the maternal depressive symptom based on the multivariate multinomial regression model

	Moderate-stable		Moderate-increasing		High-decreasing	
Determinants	aOR	95% CI	aOR	95% CI	aOR	95% CI
Ethnicity: non-Caucasian	1.91*	1.03–3.54	2.37	0.77–7.35	3.87*	1.30–11.53
Past depression: yes	3.09***	1.97–4.85	4.67***	2.01–10.82	8.57***	3.29–22.30
Stress level: high	3.16***	2.05–4.85	4.28***	1.85–9.87	18.96***	5.99–59.96
Smoking: yes	1.72	0.82–3.59	1.63	0.45–5.92	3.58*	1.17–10.97

Reference group is the low-stable trajectory group; variables included in the model: ethnicity, past depression, income, stress level, and smoking

*aOR* adjusted odds ratio, *CI* confidence interval

*p-value < 0.05. ***p-value < 0.001; *n* = 607 after removing the missing data in the covariates

Results of multinomial regression for anxiety groups showed that compared to the very low-stable group, the only variable associated with the low-stable group was the high stress level. Higher stress level, history of depression, and low income were associated with the moderate-stable group, relative to the very low-stable group (refer to Table 3).

**Table 3:** Sociodemographic, psychosocial, and behavioral predictors of the latent trajectory group membership for the maternal anxiety symptom based on the multivariate multinomial regression model

	Low-stable		Moderate-stable	
	aOR	95% CI	aOR	95% CI
Income				
<$20,000/assistance	2.69	0.59–12.29	6.00*	1.2528.81
$20,000 – $40,000	0.75	0.378–1.51	1.47	0.67–3.24
$40,000 - $60,000	1.25	0.64–2.43	2.87**	1.34–6.16
Past depression: yes	1.70	0.87–3.36	6.01***	2.91–12.40
Stress level: high	2.91**	1.49–5.68	4.83***	2.34–9.98

The reference group is the very low-stable trajectory group; variables included in the model: ethnicity, past depression, income, and stress level

*aOR* adjusted odds ratio, *CI* confidence interval

*p-value < 0.05. **p-value < 0.01. ***p-value < 0.001; *n* = 596 after removing the missing data in the covariates

## Discussion

We identified four trajectory groups of maternal depression and three trajectory groups of maternal anxiety. The four depression trajectory groups were: low-stable (35.0%), moderate-stable (54.0%), moderate-increasing (5.2%), and high-decreasing (5.9%). Women who belonged to the low-stable and moderate-stable had EPDS scores that were below the cutoff point of clinical significance throughout the period of follow-up. Our findings are consistent with other studies, such as van der Waerden et al. [10], Denckla et al. [11], Campbell et al. [35] and Luoma et al. [33], which identified trajectory groups with no symptoms, low symptoms, and/or moderate symptoms of depression that were relatively stable across the period of follow-up and had the highest proportion of participants.

Like our high-decreasing group, van der Waerden and colleagues identified a prenatal group (5%) with high symptoms during pregnancy that decreased after giving birth and increased again between 36 and 60 months postpartum [10]. The slight increase in EPDS scores seen between the 36th and 60th months postpartum could possibly be related to a subsequent pregnancy, although this information was not readily available from the FIP data. Whereas previous studies concluded a small group of high symptoms that were relatively stable over time (also referred to as “chronic”) [10, 11, 33, 35], groups with high depressive symptoms in the present study displayed more fluctuation over time. Nonetheless, caution is required when comparing our results to these studies because of the variation in the period of follow-up and the tool used to assess depression.

We also identified three anxiety trajectory groups, very low-stable (13.0%), low-stable (58.1%), and moderate-stable (29.0%). Bayrampour et al. [16], documented five trajectory groups of maternal anxiety among 1445 women in Canada, who were followed from pregnancy to 1 year postpartum. Around 70% of the women in our sample experienced very low or low anxiety symptoms throughout the period of follow-up, which is comparable to Bayrampour’s results [16]. Almost a third of our sample had moderate-high anxiety symptoms that were stable across pregnancy to 5 years postpartum, whereas Bayrampour et al., concluded two groups of high anxiety symptoms that varied over time; antepartum and postpartum groups. They also concluded a very small group (1.5%) with chronic anxiety symptoms [16]. Likewise, the three trajectories (decreasing, increasing, and transient groups) with moderate-high anxiety symptoms in Barthel’s study showed fluctuation across the perinatal period [19].

We identified maternal risk factors associated with maternal depression and/or anxiety trajectory groups; high stress level and history of depression consistently predicted groups with moderate to high depressive or anxiety symptoms, and as the severity of symptom increases the magnitude of the impact of these factors increase, suggesting a dose-response relationship. As Britton [2] documented, women who have experienced depression in the past are vulnerable to both depression and anxiety during pregnancy, postpartum, as well as to persistent symptoms that extend well beyond the perinatal period. The present study concludes that stress is a major determinant of women’s mental health, especially during childbearing period, which is also consistent with van der Waerden and Bayrampour’s results [10, 16]. Researchers report that stress can invoke hormonal changes including increased activity of the HPA axis, and reduced levels of norepinephrine [36, 37], which can trigger maternal depressive and/or anxiety symptoms. Being non-Caucasian emerged as a significant predictor of trajectory groups with high depressive symptoms, which is consistent with van der Waerden et al.’s [10], conclusions. Low income has been documented to increase the risk of both depression and anxiety [2, 38], as it was for the trajectory group with moderate anxiety symptoms but, this factor was not significantly associated with distinct trajectory groups for depressive symptoms. This could be related to the small sample size of groups with high depressive symptoms, and the small number of low-income participants in our sample. The only behavioural factor that significantly predicted high depressive symptoms groups was tobacco use, which is in keeping with the literature that showed a significant association between prenatal smoking and perinatal depression [39–41].

Strengths of the present study are the longitudinal nature and the repeated assessments during pregnancy, postpartum and up to 5 years postpartum and the use of validated screening tools for maternal depression and anxiety. Our sample included women who may be at low risk of maternal mental disorders (mostly Caucasian women with relatively high socioeconomic status), and thus the generalizability of our results may be limited to women of similar circumstance. The high attrition rates, particularly of those who could be at high risk of being depressed or anxious (such as non-Caucasian women, women with low socioeconomic status), which may have led to the underestimation of the severity of these disorders, and may have affected the significance and magnitude of association with maternal risk factors. We considered baseline covariates in our analysis, as information about some time varying covariates (stress, income, etc.) was not collected at each assessment wave. The size of some trajectory groups was small, which may have affected the power to detect true associations and their precision in relation to some of the risk factors.

## Conclusion

Our results have shown that while some women with perinatal mental health symptoms may recover quickly, for others, these symptoms may be chronic. This heterogeneity of symptoms may necessitate multiple assessments for depressive and anxiety during pregnancy and the postpartum period, and beyond to recognize women at high risk of ongoing depression or anxiety. Furthermore, recognizing these women may allow for preventative and treatment interventions, which may alter symptom progress over time. Healthcare providers for women in the perinatal period should inquire about past psychiatric illness, as it appears to be a major predictor of perinatal depression and anxiety [2, 38, 42], and about stress levels, particularly during pregnancy and around birth, which can be major transitional periods in a woman’s life. Public health interventions that target some of the modifiable risk factors (e.g. smoking) specifically designed for women in the perinatal period may reduce the burden of these illnesses at the population level. Further research is recommended to examine the evolution of depressive and anxiety symptoms over longer periods and among different populations, particularly high-risk populations of women.

## Additional files

Additional file 1: Model fit indices for maternal depression trajectories with 2–6 groups. Provide details of the model selection criteria used to select the best number and shape of maternal depression trajectory groups. (DOCX 18 kb)

Additional file 2: Diagnostic statistics for judging model selection for trajectories of maternal depression. Provide the details of the model diagnostics used for judging the final maternal depression trajectory model adequacy. (DOCX 18 kb)

Additional file 3: EPDS scores summarized by time and trajectories of maternal depression. A table summarizing the total EPDS scores over time of participants included in each maternal depressive trajectory group. (DOCX 22 kb)

Additional file 4: Maternal depression and anxiety trajectories parameter estimates (standard errors) [*n* = 615]. Provide details of the maternal depression and anxiety trajectories parameter estimates, as well as the number and percentages of participant included in each trajectory group in models with and without the dropout SAS extension. (DOCX 22 kb)

Additional file 5: Model fit indices for maternal anxiety trajectories with 2–6 groups. Provide details of the model selection criteria used to select the best number and shape of maternal anxiety trajectory groups. (DOCX 18 kb)

Additional file 6: Diagnostic statistics for judging model selection for trajectories of perinatal anxiety. Provide details of the model diagnostics used for judging the final maternal anxiety trajectory model adequacy. (DOCX 18 kb)

Additional file 7: EPDS-A scores summarized by time and trajectories of maternal anxiety. A table summarizing the EPDS-A scores over time of participants included in each maternal anxiety trajectory group. (DOCX 21 kb)

## Abbreviations

aOR: Adjusted odds ratio
BIC: Bayesian Information Criterion
CI: Confidence interval
CNORM: Censored normal distribution
EPDS: Edinburgh Postnatal Depression Scale
EPDS-A: Edinburgh Postnatal Depression Scale-Anxiety subscale
FIP: The Feeling in Pregnancy and Motherhood study
