# Supplementary material for: Trajectories of maternal depressive and anxiety symptoms from pregnancy to five years postpartum and their prenatal predictors
Source: BMC Pregnancy Childbirth. 2019 Jan 14;19:26. doi: 10.1186/s12884-019-2177-y (PMC6332639; doi:10.1186/s12884-019-2177-y)
Supplement: Supplementary file 2 — Diagnostic statistics for judging model selection for trajectories of maternal depression. Provide the details of the model diagnostics used for judging the final maternal depression trajectory model adequacy. (DOCX 18 kb) [file 12884_2019_2177_MOESM2_ESM.docx]

**Additional file** **2**. *Diagnostic statistics for judging model selection for trajectories of maternal depression.*

| Group | Average posterior probability | Proportion of sample assigned to group | Estimated group probability | odds of correct classification | CI for group membership probability |
| --- | --- | --- | --- | --- | --- |
| 1 | 0.84 | 0.35 | 0.36 | 9.43 | 0.29- 0.42 |
| 2 | 0.84 | 0.54 | 0.52 | 4.88 | 0.48-0.56 |
| 3 | 0.83 | 0.05 | 0.06 | 75.68 | 0.04- 0.08 |
| 4 | 0.91 | 0.06 | 0.06 | 148.62 | 0.05-0.08 |
