# Supplementary material for: Trajectories of maternal depressive and anxiety symptoms from pregnancy to five years postpartum and their prenatal predictors
Source: BMC Pregnancy Childbirth. 2019 Jan 14;19:26. doi: 10.1186/s12884-019-2177-y (PMC6332639; doi:10.1186/s12884-019-2177-y)
Supplement: Supplementary file 3 — EPDS scores summarized by time and trajectories of maternal depression. A table summarizing the total EPDS scores over time of participants included in each maternal depressive trajectory group. (DOCX 22 kb) [file 12884_2019_2177_MOESM3_ESM.docx]

**Additional file 3.** *EPDS scores summarized by time and trajectories of maternal depression.*

|  | Time of assessment | | | | |
| --- | --- | --- | --- | --- | --- |
|  | Early  pregnancy | Late  Pregnancy | Early  Postpartum | 36 months postpartum | 60 months postpartum |
| Low stable  Number of subjects  No. (%) depressed (EPDS>12)  mean (SD) | 215  1 (0.47)  3.37 (2.42) | 209  0 (0.00)  2.82 (2.11) | 212  1 (0.47)  2.76 (2.16) | 139  0 (0.00)  1.95 (1.76) | 129  0 (0.00)  2.42 (2.16) |
| Moderate stable  Number of subjects  No. (%) depressed (EPDS>12)  mean (SD) | 332  31 (9.34)  7.07 (3.36) | 324  12 (3.70)  6.99 (2.98) | 319  8 (2.51)  6.29 (3.17) | 173  4 (2.31)  5.64 (3.34) | 158  4 (2.53)  5.74 (3.17) |
| Moderate-increasing  Number of subjects  No. (%) depressed (EPDS>12)  mean (SD) | 32  4 (12.50)  9.09 (3.09) | 32  8 (25.00)  9.59 (4.51) | 32  18 (56.25)  14.06 (4.78) | 13  9 (69.23)  14.00 (2.83) | 10  5 (50.00)  11.70 (5.12) |
| High-decreasing  Number of subjects  No. (%) depressed (EPDS>12)  mean (SD) | 36  27 (75.00)  16.11 (4.71) | 36  27 (75.00)  15.53 (4.51) | 29  6 (20.69)  9.21 (5.05) | 12  1 (8.33)  7.50 (3.40) | 11  4 (36.36)  11.18 (3.66) |

Notes: EPDS= Edinburgh Postnatal Depression Scale; SD, standard deviation.
