# Supplementary material for: Trajectories of maternal depressive and anxiety symptoms from pregnancy to five years postpartum and their prenatal predictors
Source: BMC Pregnancy Childbirth. 2019 Jan 14;19:26. doi: 10.1186/s12884-019-2177-y (PMC6332639; doi:10.1186/s12884-019-2177-y)
Supplement: Supplementary file 4 — Maternal depression and anxiety trajectories parameter estimates (standard errors) [n = 615]. Provide details of the maternal depression and anxiety trajectories parameter estimates, as well as the number and percentages of participant included in each trajectory group in models with and without the dropout SAS extension. (DOCX 22 kb) [file 12884_2019_2177_MOESM4_ESM.docx]

**Additional file 4.** *Maternal depression and anxiety trajectories parameter estimates (standard errors) [n=615].*

| Group | Intercept | Linear | Quadratic | Cubic | Group membership |
| --- | --- | --- | --- | --- | --- |
| Depression trajectories | | | | | |
| *Model with dropout extension* | | | | | |
| Low stable | 3.73*** (0.34) | -0.36*** (0.09) | - | - | 35.76%***  (4.64) |
| Moderate stable | 7.95*** (0.34) | -0.55*** (0.09) | - | - | 51.81%***  (4.22) |
| Moderate-increasing | 14.62*** (3.82) | -10.13 (5.78) | 5.09* (2.41) | -0.64* (0.29) | 6.06%**  (1.91) |
| High-decreasing | 7.28 (3.78) | 15.19** (5.12) | -7.63*** (2.01) | 0.94*** (0.23) | 6.37%***  (1.44) |
| *Model without dropout extension* | | | | | |
| Low stable | 3.68*** (0.38) | -0.40*** (0.10) | - | - | 32.98%***  (5.02) |
| Moderate stable | 7.81*** (0.40) | -0.61*** (0.09) | - | - | 51.14%**  (4.54) |
| Moderate-increasing | 11.29*** (3.38) | -1.79 (5.23) | 0.44 (2.34) | -0.02 (0.29) | 11.05%***  (3.75) |
| High-decreasing | 11.65** (4.22) | 8.34 (6.44) | -4.02 (2.94) | 0.44 (0.37) | 4.83%***  (1.19) |
| Anxiety trajectories |  |  |  |  |  |
| *Model with dropout extension* | | |  |  |  |
| Very low-stable | 1.64**  (0.51) | -1.11**  (0.40) | 0.16*  (0.07) | - | 13.71%***  (2.56) |
| Low-stable | 3.69***  (0.25) | -0.83***  (0.18) | 0.10***  (0.03) | - | 56.12%***  (3.35) |
| Moderate-stable | 5.43***  (0.19) | -0.34***  (0.06) | - | - | 30.17%***  (3.73) |
| *Model without dropout extension* | |  |  |  |  |
| Very low-stable | 1.76**  (0.56) | -1.15*  (0.47) | 0.15  (0.08) | - | 14.00%***  (3.43) |
| Low-stable | 3.61***  (0.28) | -0.77***  (0.18) | 0.09**  (0.03) | - | 55.00%***  (3.43) |
| Moderate-stable | 5.45***  (0.20) | -0.37***  (0.05) | - | - | 30.98%***  (4.31) |

*Notes:* **p-value*<0.05. ***p-value*<0.01. ****p-value*<0.001
