# Supplementary material for: Trajectories of maternal depressive and anxiety symptoms from pregnancy to five years postpartum and their prenatal predictors
Source: BMC Pregnancy Childbirth. 2019 Jan 14;19:26. doi: 10.1186/s12884-019-2177-y (PMC6332639; doi:10.1186/s12884-019-2177-y)
Supplement: Supplementary file 5 — Model fit indices for maternal anxiety trajectories with 2–6 groups. Provide details of the model selection criteria used to select the best number and shape of maternal anxiety trajectory groups. (DOCX 18 kb) [file 12884_2019_2177_MOESM5_ESM.docx]

**Additional file 5.** *Model fit indices for maternal anxiety trajectories with 2-6 groups.*

| Number of groups | BIC(n=2453) | Probability of being the correct model | BIC (n=615) | Probability of being the correct model |
| --- | --- | --- | --- | --- |
| 2 | -5645.8 | 0.0000 | -5634.7 | 0.0000 |
| 3 | **-5614.9** | **0.9999** | **-5598.3** | **0.9219** |
| 4 | -5624.5 | 0.0001 | -5602.4 | 0.0167 |
| 5 | -5628.7 | 0.0000 | -5601.1 | 0.0613 |
| 6 | -5644.6 | 0.0000 | -5611.4 | 0.0000 |

*Notes*: BIC, Bayesian information criterion; n=2,453, the total number of assessments used in model estimation across persons and time; n=615, the number of individuals in the model estimation.
