# Supplementary material for: Trajectories of maternal depressive and anxiety symptoms from pregnancy to five years postpartum and their prenatal predictors
Source: BMC Pregnancy Childbirth. 2019 Jan 14;19:26. doi: 10.1186/s12884-019-2177-y (PMC6332639; doi:10.1186/s12884-019-2177-y)
Supplement: Supplementary file 6 — Diagnostic statistics for judging model selection for trajectories of perinatal anxiety. Provide details of the model diagnostics used for judging the final maternal anxiety trajectory model adequacy. (DOCX 18 kb) [file 12884_2019_2177_MOESM6_ESM.docx]

**Additional file 6.** *Diagnostic statistics for judging model selection for trajectories of maternal anxiety*.

| Group | Average posterior probability | Proportion of sample assigned to group | Estimated group probability | odds of correct classification | CI for group membership probability |
| --- | --- | --- | --- | --- | --- |
| 1 | 0.88 | 0.13 | 0.14 | 46.16 | 0.11-0.16 |
| 2 | 0.87 | 0.58 | 0.56 | 5.23 | 0.53-0.59 |
| 3 | 0.87 | 0.29 | 0.30 | 15.49 | 0.26-0.34 |
