# Supplementary material for: Trajectories of maternal depressive and anxiety symptoms from pregnancy to five years postpartum and their prenatal predictors
Source: BMC Pregnancy Childbirth. 2019 Jan 14;19:26. doi: 10.1186/s12884-019-2177-y (PMC6332639; doi:10.1186/s12884-019-2177-y)
Supplement: Supplementary file 7 — EPDS-A scores summarized by time and trajectories of maternal anxiety. A table summarizing the EPDS-A scores over time of participants included in each maternal anxiety trajectory group. (DOCX 21 kb) [file 12884_2019_2177_MOESM7_ESM.docx]

**Additional file 7.** *EPDS-A scores summarized by time and trajectories of maternal anxiety.*

|  | Time of assessment | | | | |
| --- | --- | --- | --- | --- | --- |
| Trajectory | Early  pregnancy | Late  Pregnancy | Early  Postpartum | 36 months postpartum | 60 months postpartum |
| Very low-stable  Number of subjects  No. (%) depressed (EPDS-A>4)  mean (SD) | 80  0 (0.00)  0.99 (1.07) | 78  0 (0.00)  0.76 (0.90) | 80  0 (0.00)  0.50 (0.76) | 64  0 (0.00)  0.48 (0.82) | 64  1 (1.56)  0.73 (1.00) |
| Low-stable  Number of subjects  No. (%) anxious (EPDS-A>4)  mean (SD) | 357  62 (17.37)  2.93 (1.55) | 349  29 (8.31)  2.50 (1.41) | 348  26 (7.47)  2.22 (1.53) | 201  6 (2.99)  2.02 (1.32) | 178  13 (7.30)  2.28 (1.45) |
| Moderate-stable  Number of subjects  No. (%) anxious (EPDS-A>4)  mean (SD) | 178  128 (71.91)  5.30 (1.49) | 173  105 (60.69)  4.84 (1.53) | 164  80 (48.78)  4.55 (1.72) | 72  28 (38.89)  4.10 (1.70) | 66  26 (39.39)  4.02 (1.84) |

*Notes:* EPDS-A, 3-item anxiety subscale; SD, standard deviation.
